# Maternal use of probiotics during pregnancy and effects on their offspring’s health in an unselected population

**DOI:** 10.1007/s00431-015-2618-1

**Published:** 2015-08-30

**Authors:** Nicole Rutten, Anne Van der Gugten, Cuno Uiterwaal, Arine Vlieger, Ger Rijkers, Kors Van der Ent

**Affiliations:** Department of Pediatric Pulmonology and Allergology, Wilhelmina Children’s Hospital, University Medical Center Utrecht, Room KH.01.419.0, PO Box 85090, Utrecht, 3508 AB The Netherlands; Department of Pediatrics, St. Antonius Hospital, PO Box 2500, Nieuwegein, 3430 EM The Netherlands; Julius Center for Health Sciences and Primary Care, University Medical Center Utrecht, PO Box 85500, Utrecht, 3508 GA The Netherlands; Laboratory of Medical Microbiology and Immunology, St. Antonius Hospital, PO Box 2500, Nieuwegein, 3430 EM The Netherlands; Department of Sciences, University College Roosevelt, PO Box 94, Middelburg, 4330 AB The Netherlands

**Keywords:** Probiotics, Behavioural patterns, Pregnancy, Health effects

## Abstract

Probiotics are used by women in the perinatal period and may improve balance of microbiota, with possible health benefits for both mother and baby. Characteristics and (health) behaviour patterns of mothers using probiotics during pregnancy, and health effects on their offspring, were investigated. Differences between mothers using probiotics during pregnancy and those who did not, were assessed. In total, 341 out of 2491 (13.7 %) mothers reported use of probiotics during pregnancy. There were no significant differences in maternal features (gestation, age, ethnicity, education) between users and non-users. Logistic regression analyses showed that consumption of probiotics was significantly associated with use of homeopathic products [odds ratio (OR) 1.65, 95 % confidence interval (CI) 1.17–2.33, *p* = 0.005], maternal history of smoking (OR 1.72, 95 % CI 1.25–2.37, *p* = 0.001) and paternal history of smoking (OR 1.39, 95 % CI 1.01–1.89, *p* = 0.05). Common disease symptoms during the first year of life in the offspring did not differ between both groups.

*Conclusion*: The use of probiotics or other health-related products without doctor’s prescription during pregnancy might point to compensation for types of less favourable behaviour. Probiotic use during pregnancy does not seem to induce positive health effects in the offspring in an unselected population.

**Table:** 

**What is Known:** • *Aberrant microbiota compositions have been detected during critical periods when early programming occurs including pregnancy and early neonatal life.* • *Probiotics modulate intestinal microbiota composition and are associated with positive health effects.*
**What is New:** • *The use of probiotics or other health-related products without doctor’s prescription during pregnancy is associated with and might point to compensation for types of less favourable behaviour.* • *Probiotic use during pregnancy does not induce positive health effects in the offspring in this unselected population.*

## Introduction

Adequate nutrition is of major importance for one’s health and well-being, especially during preconception and pregnancy [3, 5, 17, 18, 21]. Women become more aware of the health aspects of nutrition during pregnancy and seek for more nutrition-related information. Compared to the period before conception and pregnancy, pregnant women are more interested in healthy food and may be more receptive to behaviour change and lifestyle interventions [2, 26, 32, 33].

The increased nutrient requirements during pregnancy are mostly covered by a balanced diet, but dietary supplements are often taken to improve maternal or foetal health status [1]. Maternal (health) behaviour and micronutrient status during pregnancy have been linked to the health status of the child [4, 5, 17, 19, 21]. Moreover, aberrant microbiota compositions have been detected during critical periods when early programming occurs, including pregnancy and early neonatal life [17, 19, 25]. Manipulation of the maternal microbiota composition through the use of probiotics may have subsequent consequences for the health of the offspring, as the presence of bacteria in human milk implicates that modulation of maternal gut microbiota during pregnancy and lactation could have an effect on infant health [10, 13, 14, 23]. Improvement of maternal intestinal microbiota composition, relief of possible gastrointestinal complaints, reduced infant’s risk of developing atopic dermatitis, atopic sensitization and gastrointestinal symptoms as well as changes in foetal and infant’s growth have been reported as positive health effects of probiotics [9, 12, 19, 22, 24]. In western societies, a substantial percentage of pregnant women appear to use probiotic supplements [4, 7]. Because of the potential positive effects for the health of the woman and her neonate, pregnancy is an opportune time for probiotic use. Both beliefs and knowledge seem to strongly affect the mother’s behaviour [6]. Review of the literature shows that ingestion of probiotics (combination of strains of *Lactobacillus* and *Bifidobacterium*) for a limited period of time during (late) pregnancy appears to be low risk, as it does not increase the rate of adverse pregnancy outcomes and seems to be well tolerated [30].

The aim of our study was to investigate characteristics and health behaviour patterns of mothers who use probiotics during pregnancy. As a secondary aim, we studied the effects of maternal use of probiotics on the offspring’s health during the first year of life.

## Materials and methods

### Study design and study population

Subjects of the present study were mothers, with their child, participating in the ongoing Wheezing Illnesses Study Leidsche Rijn (WHISTLER) study. WHISTLER is a large prospective birth cohort study that started in December 2001 [15]. Baseline pre-pregnancy data of these parents were available from the Utrecht Health Project [11]. At the infant’s age of 3–8 weeks, information on pre- and post-natal risk factors is obtained by questionnaires and the infant’s birth weight and height, as well as gestational age and gender are recorded at an outpatient visit. Health parameters during the infant’s first year of life are followed in the WHISTLER study through linkage with the computerized medical files recorded by general practitioners.

### Definitions of outcomes

General characteristics and behaviour patterns of the mother (and father) were extracted from the WHISTLER database. History of smoking was defined as smoking *ever*, prior to pregnancy (without limitation in months/years ago). A positive history of parental allergy was defined as questionnaire-reported allergy to pollen, house dust mite, pets or food. Maternal higher education was defined as higher vocational or university education. Maternal paid occupation was defined as having a paid job (yes or no) at time of completing the questionnaire.

At the visit shortly after birth, maternal use of probiotics was asked as follows: Did you use probiotics during pregnancy, either as in a probiotic milk or yoghurt product and/or probiotic-containing supplements? If yes, how many portions did you (on average) use per week? One portion was defined as use of one sachet or one capsule or use of one serving of a known probiotic-containing milk or yoghurt product. Active maternal smoking during pregnancy was considered present if the mother smoked at least one cigarette per day during pregnancy. Exposure to smoke during pregnancy was defined present when the mother smoked herself and/or if she reported being exposed to environmental cigarette smoke for at least 2 h per week. Use of supplements without doctor’s prescription was defined as maternal-reported use of at least one of the following, during the past 3 months:

1. Vitamins, minerals, iron substitutes or resistance-increasing substitutes;
2. Substitutes for other gastrointestinal complaints;
3. Substitutes against cough and cold;
4. Laxatives or sedatives.

Use of homeopathic or herbal products during the past 3 months was also recorded.

Eating fruits and/or vegetables on a regular basis was considered as a parameter for a healthy lifestyle, and the variables were defined as eating five or more pieces of fruit a week and preparing fresh vegetables seven or more times a week.

To analyze the effects of the use of probiotics on the offspring’s health, we used follow-up data from the WHISTLER study. In this study data on respiratory symptoms, disease episodes and day-care attendance are recorded during the first year of life using monthly questionnaires. Furthermore, GP diagnoses on upper respiratory tract infections, lower respiratory tract infections, gastrointestinal tract infections and constitutional eczema are recorded using International Classification of Primary Care (ICPC) codes.

### Analysis

We compared mothers that used probiotics during pregnancy to non-users. In order to assess differences between groups, chi-square tests and independent samples *t* tests were used where appropriate. For all the analyses, firstly, the univariable association with use of probiotics during pregnancy was estimated using logistic regression. Secondly, we extended to multivariable logistic regression to adjust for maternal characteristics or behaviour patterns that were significantly associated with use of probiotics or showed a trend towards significance in the univariable analysis. A cut-off *p* value of <0.30 in the univariable association was used to insert variables into the multivariate model. Results are presented as odds ratios, with 95 % confidence intervals and *p* values. Associations were considered statistically significant if *p* values were ≤0.05. Analyses were run using SPSS version 20.0 (SPSS Inc., Chicago, IL, USA).

## Results

Data of 2491 mothers were used for analysis of their lifestyle and behaviour during pregnancy. Group characteristics and maternal attitudes between mothers who did and did not use probiotics during pregnancy are shown in Table 1. Of the total group, 13.7 % of the mothers reported use of probiotics during pregnancy. The mean usage per week was 3.5 portions (ranging from 1 to 15), in which there were no differences between the first and the second half of their pregnancy (data not shown). No differences were shown between both groups for gestational age, birth weight, maternal age at time of delivery, education and ethnicity of the mother. Probiotic-using mothers more often had a history of smoking, compared to non-users (*p* = 0.003). There was also a significant association between fathers with a history of smoking and the use of probiotics by the mother (*p* = 0.01). Maternal use of probiotics during pregnancy was significantly associated with use of other supplements and substitutes without doctor’s prescription (*p* = 0.02) and the use of homeopathic products (*p* < 0.001).

**Table 1:** Characteristics of the study group

Parental characteristics	Total group	Probiotic use	Non-probiotic use	*p* value
	*n* = 2491	(13.7 %)	(86.3 %)	
Maternal age at time of delivery (mean, in years) (SD)	32.7 (3.9)	32.8 (3.7)	32.7 (4.0)	0.63^a^
Maternal weight (mean, in kg) (SD)	71.4 (12.5)	70.2 (12.4)	71.5 (12.3)	0.12^a^
Ethnicity mother (% western)	89.9	91.0	89.7	0.51^b^
Maternal higher education (%)	67.3	70.0	66.9	0.31^b^
Maternal paid occupation (%)	89.8	89.9	89.8	0.99^b^
Maternal history of smoking (prior to pregnancy *ever*) (%)	35.4	43.3	34.1	**0.003** ^b^
Maternal smoking during pregnancy (%)	6.2	7.0	6.1	0.51^b^
Maternal smoke exposure during pregnancy (%)	14.9	17.0	14.5	0.23^b^
Current smoking mother (%)	8.2	9.8	7.9	0.30^b^
Paternal history of smoking (ever) (%)	40.6	48.3	39.4	**0.01** ^b^
Current smoking father (%)	17.5	16.5	17.7	0.64^b^
Pet ownership during pregnancy (%)	39.3	41.6	38.9	0.34^b^
Use of alcohol (in general) (%)	79.6	81.2	79.4	0.50^b^
Use of substitutes/supplements without doctor’s prescription during the past 3 months (%)^c^	75.9	81.5	75.0	**0.02** ^b^
Use of homeopathic substitutes/herbal medicines during the past 3 months (%)	22.3	30.5	20.9	**<0.001** ^b^
Use of fruits (5 or more pieces a week) (%)	64.8	68.0	64.3	0.24^b^
Use of fresh vegetables (7 or more times a week) (%)	40.9	44.0	40.4	0.25^b^
Maternal allergy (%)^d^	35.1	34.1	35.2	0.71^b^
Maternal allergy (%)^e^	48.2	50.9	47.7	0.33^b^
Paternal allergy (%)^e^	43.7	41.6	44.0	0.47^b^
Children’s day-care visit during the first 6 months of life (%)	65.3	70.8	64.4	**0.03** ^b^

*p* values in bold are statistically significant

^a^ *t* test

^b^Chi-square test

^c^e.g. vitamins, minerals, iron substitutes, resistance-increasing substitutes, substitutes for other gastrointestinal complaints, substitutes against cough and cold, laxatives and sedatives

^d^Allergy to pollen, dust, house mite and pets

^e^Allergy to pollen, dust, house mite, pets, food or *other*

Table 2 shows characteristics of the offspring of both probiotic-using and non-probiotic-using mothers. In the probiotic group, mothers more frequently gave birth to a boy (*p* = 0.04). Otherwise, no differences between the groups were demonstrated.

**Table 2:** Characteristics of the study group

Infant characteristics	Total group	Probiotic use group	Non-probiotic use group	*p* value
	*n* = 2491	(13.7 %)	(86.3 %)	
Gestational age (mean, in weeks) (SD)	39.4 (1.4)	39.3 (1.5)	39.4 (1.4)	0.13^a^
Birth weight (mean, in g) (SD)	3526 (514)	3529 (539)	3525 (510)	0.87^a^
Gender (% boys within the group)	49.3	54.5	48.5	**0.04** ^b^
Siblings (% with at least one)	54.0	50.9	54.5	0.22^b^
Upper respiratory tract infections^c^ (%)	46.9	46.6	47.0	0.88^b^
Lower respiratory tract infections^c^ (%)	9.0	11.0	8.6	0.15^b^
Gastrointestinal tract infections^c^ (%)	16.8	17.3	16.7	0.79^b^
Constitutional eczema^c^ (%)	12.9	14.3	12.7	0.39^b^

*p* values in bold are statistically significant

^a^ *t* test

^b^Chi-square test

^c^During the first year of life, doctor’s diagnosis

The results of the multivariable analysis on the association between the use of probiotics and other parental characteristics are shown in Table 3. The use of probiotics during pregnancy was increased in mothers who reported use of homeopathic substitutes or herbal medicines [odds ratio (OR) 1.65, 95 % confidence interval (CI) 1.17–2.33, *p* = 0.005]. Use of probiotics by the mother was also significantly associated with a higher frequency of history of smoking of both mother (OR 1.72, 95 % CI 1.25–2.37, *p* = 0.001) and father (OR 1.39, 95 % CI 1.01–1.89, *p* = 0.05).

**Table 3:** Associations between parental characteristics and use of probiotics during pregnancy

	Multivariable analysis	
	OR (95 % CI)	*p* value
Maternal history of smoking (prior to pregnancy *ever*)	1.72 (1.25–2.37)	**0.001**
Maternal smoke exposure during pregnancy	1.06 (0.67–1.70)	0.79
Use of substitutes/supplements without doctor’s prescription during the past 3 months^a^	1.05 (0.70–1.58)	0.82
Use of homeopathic substitutes/herbal medicines during the past 3 months	1.65 (1.17–2.33)	**0.005**
Use of fruits (5 or more pieces a week)	1.14 (0.82–1.60)	0.44
Use of fresh vegetables (7 or more times a week)	0.96 (0.70–1.32)	0.81
Paternal history of smoking (ever)	1.39 (1.01–1.89)	**0.05**
Maternal higher education	1.27 (0.88–1.83)	0.20
Children’s day-care visit during the first 6 months of life	1.31 (0.92–1.88)	0.13

*p* values in bold are statistically significant

^a^See Table 1 for definition

Table 4 shows the results of the multivariable analysis on the association of maternal use of probiotics during pregnancy and offspring’s disease symptoms during the first year of life. These symptoms did not differ between infants from probiotic-using mothers and non-probiotic-using mothers in this population.

**Table 4:** Associations between maternal use of probiotics during pregnancy and infant characteristics

	Multivariable analysis	
	OR (95 % CI)	*p* value
Upper respiratory tract infections^a^	0.97 (0.77–1.22)	0.79
Lower respiratory tract infections^a^	1.31 (0.90–1.91)	0.16
Gastrointestinal tract infections^a^	1.03 (0.76–1.40)	0.86
Constitutional eczema^a^	1.15 (0.82–1.60)	0.42

^a^During the first year of life, doctor’s diagnosis

## Discussion

This study shows that about one out of seven mothers in our population used probiotics during pregnancy. Use of probiotics during pregnancy was independently associated with use of homeopathic products and with a history of smoking of both mother and father.

To our knowledge, to date, no other studies analyzed the association between maternal use of probiotic supplements and other behaviour patterns during pregnancy. The number of mothers that reported consumption of probiotics during pregnancy in our cohort corresponds reasonably with previous estimates [4, 7]. In our study, mothers that used probiotics during pregnancy were not characterized by specific maternal features (gestation, age, ethnicity, education) compared to mothers that did not use probiotics during pregnancy, although the literature shows that generally the adequacy of micronutrient intake during pregnancy is related to environmental, cultural and demographic variables [4, 5, 16].

To many, probiotics, homeopathic products and nutritional and dietary supplements belong to the category of complementary medicines. Pregnancy is a time to become more aware of a healthy lifestyle including healthy nutrition. Taking any form of supplement may be part of such a (change in) lifestyle. We hypothesized that next to the health-promoting properties, that are suggested for probiotics, mothers may use probiotics during pregnancy to compensate for adverse (prior) habits of themselves or their partners, for instance smoking.

We showed comparable disease symptoms during the first year of life in the offspring from probiotic-using and non-probiotic-using mothers. Reviews and a meta-analysis demonstrated that current evidence on the effects of probiotics on the offspring’s health is fairly inconclusive [8, 25, 27]. Our data do not add evidence for a beneficial effect.

The main strength of this study was the sample size which was large enough to estimate correlates of probiotic use during pregnancy. Our data have been prospectively documented, and all extensive parental characteristics and behaviour patterns could be aggregated from the database. Former studies of our group have demonstrated that the results may be generalized to other populations [28].

However, there are also some limitations. Use of supplements, and especially probiotics, may have been underreported due to non-recall or format of the questions, as has been reported in the literature [31]. Nevertheless, we cannot conceive that non-recall of probiotic use would be related to use of other supplements or history of smoking and, therefore, is unlikely to have caused real bias.

Also, neither the type of probiotic supplement nor the regularity of intake was specified and we were not able to investigate the use of probiotics by the mothers before and after pregnancy, which would have helped to discriminate mothers based on their using habits. There is emerging evidence that the effect of probiotics is strain specific and that timing, administration route and the applied dose do affect the outcomes. We consider the current reported conclusions valid and reliable because of the standardized manner of data collection, correction for potential confounders and presence of the unselected population. Moreover, we consider our population size sufficiently large to render our results statistically robust.

Thirdly, as reported earlier, in the study population of the Utrecht Health Project and WHISTLER study, a vast percentage of participants completed higher vocational or university education [20, 29]. High socio-economic status and ethnicity might have played a role in parents’ decision to participate, which results in a not entirely unselected study population. This effect will be mediated in the population but has to be taken into account when results are generalized to lower class (young) families.

## Conclusion

This study shows that about one out of seven mothers in our population use probiotics during pregnancy. Probiotic-using mothers are not characterized by specific maternal features (gestation, age, ethnicity, education) compared to non-users. Use of probiotics during pregnancy is independently associated with use of homeopathic products and with parental history of smoking. According to common doctors’ diagnosed disease symptoms in the offspring the first year of life, no differences between groups are observed. Using probiotics and/or other health-related products without doctor’s prescription during pregnancy, for their health-promoting properties, might point to compensation for types of less favourable behaviour such as parental smoking. Caregivers and people concerned with pregnant women should be aware of this effect when discussing (nutritional) behaviour.

## Abbreviations

ICPC: International Classification of Primary Care
WHISTLER: Wheezing Illnesses Study Leidsche Rijn
